# Association of Prenatal Maternal Psychological Distress With Fetal Brain Growth, Metabolism, and Cortical Maturation

**DOI:** 10.1001/jamanetworkopen.2019.19940

**Published:** 2020-01-29

**Authors:** Yao Wu, Yuan-Chiao Lu, Marni Jacobs, Subechhya Pradhan, Kushal Kapse, Li Zhao, Nickie Niforatos-Andescavage, Gilbert Vezina, Adré J. du Plessis, Catherine Limperopoulos

**Affiliations:** 1 Center for the Developing Brain, Children’s National Hospital, Washington, DC; 2 Department of Biostatistics and Study Methodology, Children’s Research Institute, Children’s National Hospital, Washington, DC; 3 Division of Neonatology, Children’s National Hospital, Washington, DC; 4 Department of Diagnostic Imaging and Radiology, Children’s National Hospital, Washington, DC; 5 Fetal Medicine Institute, Children’s National Hospital, Washington, DC

## Abstract

**Importance:**

Prenatal maternal stress is increasingly associated with adverse outcomes in pregnant women and their offspring. However, the association between maternal stress and human fetal brain growth and metabolism is unknown.

**Objective:**

To identify the association between prenatal maternal psychological distress and fetal brain growth, cortical maturation, and biochemical development using advanced 3-dimensional volumetric magnetic resonance imaging (MRI) and proton magnetic resonance spectroscopy (1H-MRS).

**Design, Setting, and Participants:**

This cohort study prospectively recruited pregnant women from low-risk obstetric clinics in Washington, DC, from January 1, 2016, to April 17, 2019. Participants were healthy volunteers with a normal prenatal medical history, no chronic or pregnancy-induced physical or mental illnesses, and normal results on fetal ultrasonography and biometry studies. Fetal brain MRI studies were performed at 2 time points between 24 and 40 weeks’ gestation.

**Exposures:**

Prenatal maternal stress, anxiety, and depression.

**Main Outcomes and Measures:**

Volumes of fetal total brain, cortical gray matter, white matter, deep gray matter, cerebellum, brainstem, and hippocampus were measured from 3-dimensional reconstructed T2-weighted MRI scans. Cortical folding measurements included local gyrification index, sulcal depth, and curvedness. Fetal brain *N*-acetylaspartate, creatine, and choline levels were quantified using 1H-MRS. Maternal stress, depression, and anxiety were measured with the Perceived Stress Scale (PSS), Edinburgh Postnatal Depression Scale (EPDS), Spielberger State Anxiety Inventory (SSAI), and Spielberger Trait Anxiety Inventory (STAI).

**Results:**

A total of 193 MRI studies were performed in 119 pregnant women (67 [56%] carrying male fetuses and 52 [44%], female fetuses; maternal mean [SD] age, 34.46 [5.95] years) between 24 and 40 gestational weeks. All women were high school graduates, 99 (83%) were college graduates, and 100 (84%) reported professional employment. Thirty-two women (27%) had positive scores for stress, 31 (26%) for anxiety, and 13 (11%) for depression. Maternal trait anxiety was associated with smaller fetal left hippocampal volume (STAI score: –0.002 cm^3^; 95% CI, –0.003 to –0.0008 cm^3^; *P* = .004). Maternal anxiety and stress were associated with increased fetal cortical gyrification in the frontal lobe (β for SSAI score: 0.004 [95% CI, 0.001-0.006; *P* = .002]; β for STAI score: 0.004 [95% CI, 0.002-0.006; *P* < .001]; β for PSS score: 0.005 [95% CI, 0.001-0.008; *P* = .005]) and temporal lobe (β for SSAI score: 0.004 [95% CI, 0.001-0.007; *P* = .004]; β for STAI score: 0.004 [95% CI, 0.0008-0.006; *P* = .01]). Elevated maternal depression was associated with decreased creatine (EPDS score: –0.04; 95% CI, –0.06 to –0.02; *P* = .005) and choline (EPDS score: –0.03; 95% CI, –0.05 to –0.01; *P* = .02) levels in the fetal brain.

**Conclusions and Relevance:**

This study found that the prevalence of maternal psychological distress in healthy, well-educated, and employed pregnant women was high, underappreciated, and associated with impaired fetal brain biochemistry and hippocampal growth as well as accelerated cortical folding. These findings appear to support the need for routine mental health surveillance for all pregnant women and targeted interventions in women with elevated psychological distress.

## Key Points

**Question:**

What is the association between maternal stress, anxiety, and depression and in vivo fetal brain growth, metabolism, and cerebral cortical maturation?

**Findings:**

In this cohort study of 119 pregnant women, prenatal maternal psychological distress was associated with impaired fetal hippocampal development during the late second and third trimesters of gestation and altered fetal cortical gyrification in the frontal and temporal lobes. Maternal depression was also associated with decreased choline and creatine levels in the fetal brain.

**Meaning:**

Findings from this study suggest that prenatal maternal psychological distress may have an adverse association with brain structure and biochemistry in utero in the human fetus.

## Teaser

This cohort study examines the association between prenatal maternal psychological distress and fetal brain growth, cortical maturation, and biochemical development by using magnetic resonance imaging and proton magnetic resonance spectroscopy.

## Introduction

Perinatal mental health problems are a major public health issue and are associated with detrimental and enduring consequences on maternal and child health.^1,2,3,4^ Depression and anxiety are the most common mental health problems during pregnancy, although prevalence rates vary by population characteristics, timing, and type of screening used. Previous systematic reviews have suggested that up to 18% of pregnant women experience depression, 14% to 54% experience anxiety, and many experience both.^1,5,6,7,8^ The term psychological distress is often used to encompass stress, depression, and/or anxiety that have not reached the severity of a mental disorder.^9^

Maternal mental health problems in pregnancy have been associated with an elevated risk for spontaneous abortion,^10^ preeclampsia,^11^ preterm delivery,^12^ and lower birth weight.^13^ Adverse child outcomes are increasingly reported across the spectrum of learning,^14^ behavioral^4^ and interpersonal problems, and neuropsychiatric dysfunction.^15^ Differences in human brain development have also been described in the postnatal months and years after intrauterine exposure to maternal psychological distress during pregnancy. These findings have included smaller head circumference,^13^ reduced cerebral and cerebellar gray matter volume,^16,17,18^ increased amygdala^19,20^ and decreased hippocampal volumes,^21^ and altered brain microstructure^22,23^ and connectivity.^24,25^ Furthermore, disturbances in brain biochemicals have been reported in animal studies, including reductions in *N*-acetylaspartate (NAA; a marker of neuronal integrity) in the frontal cortex and hypothalamus in early life stress–exposed mice^26,27,28^ as well as altered neurotransmitter metabolism of γ-aminobutyric acid and glutamate in the right hippocampus of pregestational stress–exposed offspring.^29^ Although a growing body of evidence finds a correlation between prenatal maternal psychological distress and neurodevelopmental dysfunction in their offspring, the association of psychological distress with fetal brain development and metabolism remains poorly understood at this time.

Identifying early modifiable risk factors for brain dysfunction is critical for developing early, individualized, and rational treatment strategies to better support fetal neurodevelopment. The successful applications of advanced magnetic resonance imaging (MRI) and proton magnetic resonance spectroscopy (1H-MRS) techniques to the living fetus^30,31,32^ provide an unprecedented opportunity to study the association between maternal psychological distress and human fetal brain development. We therefore sought to identify the associations of maternal stress, depression, and anxiety with fetal brain volumetric growth, cortical folding, and metabolism using 3-dimensional reconstructed T2-weighted MRI and 1H-MRS.

## Methods

### Study Design

Between January 1, 2016, and April 17, 2019, we prospectively recruited pregnant women into a longitudinal observational cohort study. Participants were healthy volunteers from low-risk obstetric clinics in Washington, DC. Women were eligible for inclusion if, as confirmed by their medical records, they had a normal prenatal medical history; had no chronic or pregnancy-induced physical or mental illnesses; and had normal results on screening serum tests, fetal ultrasonography, and fetal biometry studies. We excluded (1) fetuses with known or suspected congenital infection, dysmorphic features or dysgenetic lesions, or documented genetic or chromosomal abnormalities and (2) pregnant women with chronic or pregnancy-induced medical conditions (eg, autoimmune, metabolic, genetic, or psychiatric); pregnancy complications that developed after study enrollment; multiple pregnancies; self-reported licit or illicit drug abuse, smoking, or alcohol use; medications for chronic conditions (eg, enoxaparin, selective serotonin reuptake inhibitor, or levothyroxine); and contraindications to MRI (eg, metal implants or claustrophobia). Fetal brain MRI studies were performed at 2 time points between 24 and 40 weeks’ gestation. This study was approved by the institutional review board at Children’s National Hospital. Written informed consent was obtained from all participants before enrollment by a study staff person who met with each eligible patient to review the study objectives and procedures. We followed the Strengthening the Reporting of Observational Studies in Epidemiology (STROBE) reporting guideline.

### Maternal Stress, Depression, and Anxiety

 Psychometrically sound questionnaires that measure stress (Perceived Stress Scale [PSS]),^33^ anxiety (Spielberger State Anxiety Inventory [SSAI] and Spielberger Trait Anxiety Inventory [STAI]),^34^ and depression (Edinburgh Postnatal Depression Scale [EPDS])^35^ were completed on the same day as each MRI visit. These questionnaires have been widely used in pregnancy studies.^36,37,38,39,40,41^ The PSS measures the degree of stressful feelings experienced during the past month. The score range for the 10-item PSS is 0 to 40, with a score higher than 15 indicating that the perceived stress is higher than average.^37,42^ Both the SSAI (which assesses “how you feel right now”) and the STAI (which assesses “how you generally feel”) include 20 items and have a score range of 20 to 80, with a score higher than 40 indicating the presence of anxiety.^38,39^ The 10-item EPDS is designed to measure the severity of depression in the past 7 days and is commonly used during and after pregnancy.^43^ An EPDS score ranges from 0 to 30, with a score higher than 10 indicating symptoms of depression during pregnancy.^40,41^

### MRI Acquisition and Fetal Brain Reconstruction

Fetal brain T2-weighted MRI was performed using a 1.5-T scanner (Discovery MR450; GE Healthcare) and an 8-channel receiver coil. The scanning protocol included multiplanar, single-shot fast-spin echo acquisitions (echo time: 160 milliseconds; repetition time: 1100 milliseconds; flip angle: 90°; field of view: 32 cm; matrix: 256 × 192; 2-mm slice thickness). Participants were free-breathing during the MRI scanning, and the acquisition time was 2 to 3 minutes for each of the axial, sagittal, and coronal planes. Images of all 3 planes were reconstructed into a high-resolution 3-dimensional volume with a validated pipeline, a parallel slice-to-volume reconstruction method using evaluated point-spread functions for the image reconstruction from motion-corrupted stacks of 2-dimensional slices.^44^ After reconstruction, images were spatially aligned to preterm brain atlas^45^ using landmark-based rigid registration in Image Registration Toolkit. The aligned images with 0.86-mm isotropic resolution were used for the following volumetric and cortical measures.

### MRI Volumetric Analysis

Volumes of total brain, cortical gray matter, white matter, deep gray matter, cerebellum, and brainstem were automatically extracted using Draw-EM software, v1.1 (Biomedia),^46^ which has been applied in fetal brain segmentation.^47^ Automatic segmentations were further manually corrected on the basis of the image information from all 3 planes (axial, coronal, and sagittal) using ITK-SNAP software (ITK-SNAP) (Figure). Left and right hippocampi were manually delineated according to previously validated anatomical criteria.^48,49^ A neuroradiologist on our team with more than 15 years’ experience in reading fetal MRI studies (G.V.) and who was blinded to psychological distress scores assisted with anatomical localization of these brain structures on fetal MRI images. All structures were manually corrected by the same rater (Y.W.), and 39 scans (20%) were randomly chosen and segmented by a second rater (K.K.). Both raters had more than 5 years of experience in fetal MRI brain segmentation. Interrater reliabilities using intraclass correlation coefficient for all measured regions were higher than 0.95. Raters were blinded to mental scores.

**Figure.**
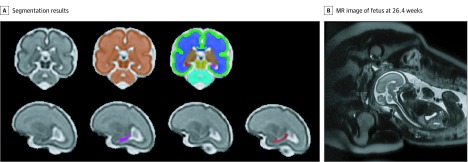
T2-Weighted Magnetic Resonance (MR) Imaging Brain Segmentation Segmentation results of total brain (orange), cortical gray matter (green), white matter (blue), deep gray matter (brown), brainstem (yellow), cerebellum (light blue), left hippocampus (purple), and right hippocampus (red) on a 3-dimensional reconstructed T2-weighted MR image of a fetus at 26.4 gestational weeks.

### Cortical Folding Measures

The inner surface of cortical gray matter (ie, the border of cortical gray matter and cerebral white matter) was used to measure the cortical folding.^50^ Four regions of interest for each hemisphere, including frontal, parietal, temporal, and occipital lobes (eFigure 1 in the Supplement), were obtained by consolidating 50 parcellated brain regions from the Draw-EM pipeline.^46^ Manual correction of the parcellated regions on cortical surface was conducted to remove holes and smooth the boundary of adjacent regions using ITK-SNAP software. For the cortical surface of each lobe, we analyzed the following measures: (1) local gyrification index, calculated as the ratio between the cortical surface area at each vertex and the corresponding area on the cerebral hull surface^51^; (2) sulcal depth, calculated as the distance from each vertex on the cortical surface to the nearest point on the cerebral hull surface^52^; and (3) curvedness, calculated as measuring the amount or intensity of surface curvature.^53^ Plots of local gyrification index, sulcal depth, and curvedness on the cortical surface of a fetus at 36.7 gestational weeks are shown in eFigure 2 in the Supplement.

### Fetal Brain Metabolism

A spectral voxel was placed in the center of the fetal brain with guidance from anatomical images acquired immediately before the spectroscopic acquisition (eFigure 3 in the Supplement). Automatic prescan that included shimming, center frequency determination, and transmit and receive gain adjustments preceded all spectroscopic acquisitions. Linewidth value obtained after automatic prescan was a measure of field homogeneity in the voxel; the smaller the linewidth, the better the homogeneity. Linewidth value of less than 9 Hz was considered acceptable to continue spectral acquisition. All data were acquired with an echo time of 144 milliseconds and a repetition time of 1500 milliseconds. Chemical shift selective water suppression sequence was used in conjunction with point resolved spectroscopy localization sequence for acquiring water-suppressed spectra.^54^ Sixteen unsuppressed water spectra averages and 192 water-suppressed spectra averages were acquired from each participant from 30 × 30 × 30-mm^3^ voxel. Spectral postprocessing included frequency and phase corrections using programs written in MATLAB (MathWorks), and the resulting spectra were analyzed using LCmodel (Stephen Provencher) with water spectrum as a reference.^55^ Metabolite concentrations were reported in institutional units (iu). Spectra that passed a visual quality check and were quantified with Cramer-Rao lower bounds with less than 20% were included for further analysis. In this study, we analyzed NAA, creatine, and choline levels in the fetal brain.

### Statistical Analysis

Analysis was performed with SAS, version 9.3 (SAS Institute Inc), and MATLAB, version R2018b (MathWorks). Participant characteristics by fetal sex were compared using 2-tailed, unpaired *t* test for continuous variables and Fisher exact test for categorical variables. Generalized estimating equations, which allowed multiple measurements for each participant, were used to assess changes in fetal brain volumes, cortical folding, metabolic measures, and psychological distress scales by gestational age and sex. Associations between maternal psychological distress and brain volumes, cortical folding, and metabolic measures were estimated using generalized estimating equations, adjusting for gestational age at the time of MRI scan and sex. Additional adjustments for maternal age, weight, educational level, employment status, and race/ethnicity as well as paternal educational level and employment status were made but did not materially change the estimates. Possible interactions between psychological distress scores and sex were also evaluated but did not have significant implications for the outcome. *P* values were adjusted for multiple testing using the false discovery rate method^56^ based on the number of outcomes, and adjusted 2-sided *P* ≤ .05 was considered statistically significant. Data analyses were performed from January 29, 2016, to July 12, 2019.

## Results

### Demographics

A diagram illustrating participant recruitment is shown in eFigure 4 in the Supplement. In this study, 21 participants completed 1 MRI study. One participant was excluded because of an abnormal MRI result, and 24 MRI scans were excluded because of severe fetal motion (19 scans [8%]) and missing maternal questionnaires (5 scans [2%]). The final data set consisted of 193 fetal MRI studies (99 at time point 1 and 94 at time point 2) with completed maternal questionnaires from 119 participants (of whom 67 [56%] were carrying male fetuses and 52 [44%], female fetuses; maternal mean [SD] age, 34.46 [5.95] years). Forty-one 1H-MRS scans (19%) were not successfully obtained, and the final 1H-MRS spectra data comprised 100 participants (171 scans). All conventional fetal MRI scans were interpreted as structurally normal. The mean (SD) gestational age at time point 1 was 28.34 (2.49) weeks and at time point 2 was 36.15 (1.80) weeks. All women were high school graduates, 99 (83%) were college graduates, and 100 (84%) reported professional employment. Participants were from a racially/ethnically diverse population; 19 (16%) were non-Hispanic black, and 71 (60%) were non-Hispanic white. Participant characteristics are summarized in Table 1.

**Table 1.** Characteristics of the Overall Study Sample and by Fetal Sex

Variable	No. (%)			*P* Value^a^
	Overall (n = 119)	Female Fetus (n = 52)	Male Fetus (n = 67)	
GA, mean (SD), wk				
At MRI study time point 1 (n = 99)	28.34 (2.49)	28.28 (2.61)	28.40 (2.42)	.81
At MRI study time point 2 (n = 94)	36.15 (1.80)	36.33 (1.80)	36.01 (1.81)	.39
Maternal characteristics				
Age, mean (SD), y	34.46 (5.95)	34.56 (5.54)	34.37 (6.29)	.86
Weight, mean (SD), kg				
At MRI study time point	75.87 (12.99)	74.56 (11.81)	76.85 (13.83)	.38
At MRI study time point 2	78.39 (12.48)	77.13 (11.22)	79.36 (13.40)	.38
Primigravida	45 (38)	21 (40)	24 (36)	.44
Primipara	61 (51)	27 (52)	34 (51)	.58
Maternal educational level				.07
Partial high school	0	0	0	
High school	5 (4)	3 (6)	2 (3)	
Partial college	12 (10)	2 (4)	10 (15)	
College graduate	37 (31)	13 (25)	24 (36)	
Graduate degree	62 (52)	32 (62)	30 (45)	
Unknown	3 (3)	2 (4)	1 (1)	
Paternal educational level				.73
Partial high school	1 (1)	0	1 (1)	
High school	14 (12)	5 (10)	9 (13)	
Partial college	13 (11)	4 (8)	9 (13)	
College graduate	29 (24)	12 (23)	17 (25)	
Graduate degree	52 (44)	25 (48)	27 (40)	
Unknown	10 (8)	6 (12)	4 (6)	
Maternal employment status				.61
Professional	100 (84)	46 (88)	54 (81)	
Skilled, clerical, or sales	4 (3)	1 (2)	3 (4)	
Semiskilled operator	3 (3)	1 (2)	2 (3)	
Unemployed or homemaker	8 (7)	2 (4)	6 (9)	
Unknown	4 (3)	2 (4)	2 (3)	
Paternal employment status				.07
Professional	90 (76)	42 (81)	48 (72)	
Skilled, clerical, or sales	7 (6)	2 (4)	5 (7)	
Semiskilled operator	4 (3)	2 (4)	2 (3)	
Unemployed or homemaker	7 (6)	0	7 (10)	
Unknown	11 (9)	6 (12)	5 (7)	
Maternal race/ethnicity				.75
Asian or Pacific Islander	8 (7)	4 (8)	4 (6)	
Non-Hispanic black	19 (16)	6 (12)	13 (19)	
Hispanic	11 (9)	6 (12)	5 (7)	
Non-Hispanic white	71 (60)	32 (62)	39 (58)	
Other or unknown	10 (8)	4 (8)	6 (9)	

Abbreviations: GA, gestational age; MRI, magnetic resonance imaging.

^a^ *P* value for difference between male and female fetuses based on 2-tailed, unpaired *t* test for continuous variables and Fisher exact test for categorical variables.

### Maternal Stress, Depression, and Anxiety

Of the 119 pregnant women, 32 (27%, with 17 [14%] carrying female fetuses and 15 [13%], male fetuses) had a positive result (measured score ≥ cutoff score) for stress, 31 (26%, with 16 [13%] carrying female fetuses and 15 [13%], male fetuses) for anxiety (24 [20%] state anxiety and 21 [18%] trait anxiety), and 13 (11%, with 8 [7%] carrying female fetuses and 5 [4%], male fetuses) for depression. Twenty-three pregnant women (19%) had a positive result for both anxiety and stress, 11 (9%) for both depression and anxiety, 12 (10%) for both depression and stress, and 10 (8%) for all 3 conditions. The correlations among maternal stress, anxiety, and depression scores were all significant, with Pearson correlation coefficients ranging from 0.65 to 0.82 (all *P* < .001). Mean maternal stress (9.97 vs 11.58), depression (3.91 vs 4.99), and anxiety (SSAI: 28.28 vs 31.33; STAI: 29.77 vs 32.62) scores did not significantly differ between mothers carrying male fetuses and those carrying female fetuses. Maternal stress scores decreased as gestational age increased (β: –0.16; 95% CI, –0.25 to –0.08; *P* < .001). However, anxiety (β for SSAI: –0.05 [95% CI, –0.26 to 0.16; *P* = .67]; β for STAI: –0.12 [95% CI, –0.26 to 0.03; *P* = .12]) and depression (β –0.01; 95% CI, –0.08 to 0.06; *P* = .75) scores were not significantly different as a function of increasing gestational age. In the subset of pregnant women who underwent MRI studies at both time points, stress (mean [SD] PSS scores: 9.51 [5.55] vs 10.99 [5.28]; *P* < .001) and trait anxiety (mean [SD] STAI scores: 29.71 [7.43] vs 31 [8.49]; *P* = .01) scores were significantly lower at time point 2 compared with time point 1 (eTable 1 in the Supplement).

### Fetal Brain Volumes, Cortical Folding, and Metabolic Measures

Male fetuses had significantly larger total brain (mean: 208.16 cm^3^ vs 197.88 cm^3^; *P* < .001), cortical gray matter (mean: 68.41 cm^3^ vs 64.44 cm^3^; *P* = .007), white matter (mean: 111.20 cm^3^ vs 105.68 cm^3^; *P* = .008), deep gray matter (mean: 18.70 cm^3^ vs 17.93 cm^3^; *P* = .002), and brainstem (mean: 4.45 cm^3^ vs 4.30 cm^3^; *P* = .01) volumes compared with female fetuses (eTable 2 in the Supplement). However, the cortical folding (local gyrification index: 1.43 for male vs 1.44 for female [*P* = .38]; sulcal depth: 1.97 mm vs 1.97 mm [*P* = .99]; curvedness: 0.22 mm^−1^ vs 0.22 mm^−1^ [*P* = .50]) and metabolic measures (NAA: 3.71 for male vs 3.50 for female [*P* = .14]; creatine: 3.06 vs 2.95 [*P* = .18]; choline: 2.45 vs 2.49 [*P* = .61]) did not differ by sex (eTable 2 in the Supplement). Mean fetal brain volumes (total brain: 17.80 cm^3^/week; cortical gray matter: 5.86 cm^3^/week; white matter: 9.14 cm^3^/week; deep gray matter: 1.33 cm^3^/week; cerebellum: 1.20 cm^3^/week; brainstem: 0.29 cm^3^/week; left hippocampus: 0.039 cm^3^/week; right hippocampus: 0.040 cm^3^/week) and cortical folding measures (local gyrification index: 0.02/week; sulcal depth: 0.16 mm/week; curvedness: 0.01 mm^−1^/week) increased with advancing gestational age (eTable 3 in the Supplement). For fetal brain metabolic measures, mean NAA (0.20/week) and creatine (0.10/week) levels increased with advancing gestational age, but not choline level (eTable 3 in the Supplement). In addition, the mean growth rates of total brain and cortical gray matter varied on the basis of sex, with significantly faster growth seen in male fetuses (total brain: 18.5 cm^3^/week [95% CI, 17.94-19.07 cm^3^/week]; cortical gray matter: 6.2 cm^3^/week [95% CI, 5.85-6.56 cm^3^/week]) compared with female fetuses (total brain: 16.93 cm^3^/week [95% CI, 16.34-17.51 cm^3^/week]; cortical gray matter: 5.5 cm^3^/week [95% CI, 5.11-5.89 cm^3^/week]) (eTable 3 in the Supplement). Volumes of the right hippocampus were larger compared with the left hippocampus in both male and female fetuses (0.03 cm^3^; 95% CI, 0.02-0.04; *P* < .001).

### Maternal Psychological Distress and Fetal Brain Volumes

Maternal trait anxiety score was negatively associated with fetal left hippocampal volume (STAI: –0.002 cm^3^; 95% CI, –0.003 to –0.0008 cm^3^; *P* = .004) (Table 2). In addition, maternal state anxiety score was negatively associated with fetal left hippocampal volume (SSAI: –0.002 cm^3^; 95% CI, –0.003 to –0.0003 cm^3^; *P* = .03), and maternal trait and state anxiety scores were negatively associated with fetal right hippocampal volume (STAI: –0.002 cm^3^ [95% CI, –0.003 to –0.0002 cm^3^; *P* = .03]; SSAI: –0.002 cm^3^ [95% CI, –0.003 to –0.0002 cm^3^; *P* = .05]) and white matter volume (STAI: –0.21 cm^3^ [95% CI, –0.40 to –0.02 cm^3^; *P* = .04]; SSAI: –0.12 cm^3^ [95% CI, –0.23 to –0.006 cm^3^; *P* = .05]), although these associations were not significant after adjusting for multiple testing.

**Table 2.** Association Between Maternal Psychological Distress and Fetal Brain Volumes^a^

Volume, cm^3^	SSAI Score		STAI Score		PSS Score		EPDS Score	
	β	*P* Value	β	*P* Value	β	*P* Value	β	*P* Value
Total brain	−0.04	.76	−0.20	.15	−0.09	.68	−0.33	.27
Cortical gray matter	0.07	.34	0.05	.50	0.07	.53	−0.10	.55
Cortical gray matter^b^	0.08	.18	0.10	.11	0.08	.37	−0.05	.71
White matter	−0.14	.07	−0.21	.04	−0.14	.37	−0.07	.78
White matter^b^	−0.12	.05	−0.09	.10	−0.13	.23	0.02	.90
Deep gray matter	0.003	.78	−0.0005	.97	0.01	.50	0.02	.39
Deep gray matter^b^	0.008	.46	0.01	.44	0.02	.29	0.04	.15
Cerebellum	0.0001	.99	−0.01	.24	0.0008	.96	−0.04	.08
Cerebellum^b^	0.001	.85	−0.0002	.97	0.004	.77	−0.03	.24
Brainstem	0.0004	.90	0.0008	.79	−0.0003	.95	−0.003	.74
Brainstem^b^	0.001	.71	0.003	.27	0.001	.74	0.004	.57
Left hippocampus	−0.002	.03	−0.002	.004^c^	−0.003	.02	−0.005	.02
Left hippocampus^b^	−0.002	.01	−0.002	.007	−0.003	.01	−0.004	.01
Right hippocampus	−0.002	.07	−0.002	.03	−0.002	.14	−0.004	.08
Right hippocampus^b^	−0.002	.05	−0.001	.08	−0.002	.15	−0.004	.08

Abbreviations: EPDS, Edinburgh Postnatal Depression Scale; PSS, Perceived Stress Scale; SSAI, Spielberger State Anxiety Inventory; STAI, Spielberger Trait Anxiety Inventory.

^a^ Sample included 119 participants and 193 scans. Results are based on generalized estimating equations, controlling for gestational age at time of magnetic resonance imaging and sex. *P* values were adjusted for multiple testing based on the false discovery rate.

^b^ Additional adjustment for total brain volume.

^c^ Statistically significant after adjusting for multiple testing.

### Maternal Psychological Distress and Fetal Cortical Folding Measures

Elevated maternal stress and anxiety scores were associated with increased local gyrification index in the frontal lobe (β for PSS: 0.005 [95% CI, 0.001-0.008; *P* = .005]; β for SSAI: 0.004 [95% CI, 0.001-0.006; *P* = .002]; β for STAI: 0.004 [95% CI, 0.002-0.006; *P* < .001]), temporal lobe (β for SSAI: 0.004 [95% CI, 0.001-0.007; *P* = .004]; β for STAI: 0.004 [95% CI, 0.0008-0.006; *P* = .01]), and global surface (β for PSS: 0.005 [95% CI, 0.002-0.008; *P* = .002]; β for SSAI: 0.003 [95% CI, 0.001-0.005; *P* = .002]; β for STAI: 0.003 [95% CI, 0.001-0.005; *P* < .001]) (Table 3). In the frontal lobe, an elevated maternal trait anxiety score was also associated with increased curvedness (β for STAI: 0.0005; 95% CI, 0.000-0.001; *P* = .03), but this association was no longer significant after adjusting for multiple testing.

**Table 3.** Association Between Maternal Psychological Distress and Fetal Cortical Folding^a^

Cortical Folding	SSAI Score		STAI Score		PSS Score		EPDS Score	
	β	*P* Value	β	*P* Value	β	*P* Value	β	*P* Value
Frontal lobe								
Local gyrification index	0.004	.002^b^	0.004	<.001^b^	0.005	.005^b^	0.005	.18
Sulcal depth	0.004	.18	0.006	.07	0.002	.66	−0.001	.88
Curvedness	0.0004	.09	0.0005	.03	0.0005	.23	0.00003	.94
Parietal lobe								
Local gyrification index	0.001	.32	0.002	.08	0.003	.19	0.0009	.73
Sulcal depth	0.004	.41	0.007	.14	0.01	.18	0.005	.65
Curvedness	−0.0002	.45	0.00007	.79	0.0001	.82	−0.0007	.27
Temporal lobe								
Local gyrification index	0.004	.004^b^	0.004	.01^b^	0.005	.02	0.006	.05
Sulcal depth	0.003	.41	0.003	.38	0.002	.77	−0.002	.87
Curvedness	0.000	.93	−0.0002	.53	−0.0002	.63	−0.0009	.07
Occipital lobe								
Local gyrification index	0.002	.11	0.002	.09	0.003	.08	0.002	.56
Sulcal depth	0.003	.37	0.003	.31	0.007	.20	0.003	.73
Curvedness	0.000	.96	−0.00006	.77	−0.0001	.78	−0.0006	.33
Global								
Local gyrification index	0.003	.002^b^	0.003	<.001^b^	0.005	.002^b^	0.004	.06
Sulcal depth	0.004	.19	0.006	.04	0.004	.35	0.003	.71
Curvedness	0.0001	.54	0.0002	.40	0.0002	.64	−0.0004	.32

Abbreviations: EPDS, Edinburgh Postnatal Depression Scale; PSS, Perceived Stress Scale; SSAI, Spielberger State Anxiety Inventory; STAI, Spielberger Trait Anxiety Inventory.

^a^ Sample included 99 participants and 142 scans. Results are based on generalized estimating equations, controlling for gestational age at time of magnetic resonance imaging and sex.

^b^ Statistically significant after adjusting for multiple testing.

### Maternal Psychological Distress and Fetal Brain Metabolism

Prenatal maternal depression score was negatively associated with creatine level (β for EPDS: –0.04; 95% CI, –0.06 to –0.02; *P* = .005) and choline level (β for EPDS: –0.03; 95% CI, –0.05 to –0.01; *P* = .02) (Table 4). In addition, NAA, creatine, and choline levels also decreased as maternal stress score increased, although these associations were no longer significant after adjusting for multiple testing (Table 4).

**Table 4.** Association Between Maternal Psychological Distress and Fetal Brain Metabolism^a^

Variable	SSAI Score		STAI Score		PSS Score		EPDS Score	
	β	*P* Value	β	*P* Value	β	*P* Value	β	*P* Value
NAA	−0.01	.24	−0.008	.39	−0.03	.04	−0.04	.05
Creatine	−0.006	.34	−0.006	.27	−0.02	.03	−0.04	.005^b^
Choline	−0.008	.19	−0.007	.22	−0.02	.04	−0.03	.02^b^

Abbreviations: EPDS, Edinburgh Postnatal Depression Scale; NAA, *N*-acetylaspartate; PSS, Perceived Stress Scale; SSAI, Spielberger State Anxiety Inventory; STAI, Spielberger Trait Anxiety Inventory.

^a^ Sample included 100 participants and 171 scans. Results are based on generalized estimating equations, controlling for gestational age at the time of magnetic resonance imaging and sex.

^b^ Statistically significant after adjusting for multiple testing.

## Discussion

This cohort study was unique in several ways. First, the participants were originally recruited to characterize in utero brain development in healthy fetuses over the second and third trimesters of pregnancy, and therefore the women had low-risk pregnancies free of common maternal or pregnancy risk factors. During the course of this study, we collected information on stress, anxiety, and depression levels. None of the pregnant participants had previously been identified as having these conditions. Second, the study population was largely composed of women with high socioeconomic and professional status and without the major psychosocial stressors usually considered as risk factors. Despite these seemingly favorable conditions, we made several unexpected findings. First, we observed a high prevalence of psychological distress in this population. Second, we found a significant association between maternal psychological distress and multiple domains of fetal brain development, including regional brain volumes, cortical gyrification, and biochemical brain development. Specifically, to our knowledge, we report for the first time that maternal anxiety may be associated with reduced fetal hippocampal volume during the late second and third trimesters of pregnancy, with the left hippocampus being more vulnerable. In addition, we showed that the fetal cortical gyrification index of the frontal and temporal lobes was altered by maternal anxiety and stress. We also reported that maternal depression score was negatively associated with creatine and choline levels in the fetal brain.

An increased risk of learning, behavioral, and neuropsychiatric problems in children and adults has been reported after early exposure to maternal mental distress.^4,14,15^ Brain imaging studies have suggested that maternal depression and anxiety in the second trimester are associated with decreased gray matter density^16^ and cortical thinning in young children, especially in the frontal and temporal lobes.^17,18,57^ Prenatal stress and depression have also been associated with alterations in limbic and frontal white matter microstructures.^18,22^ Infants exposed to prenatal maternal anxiety have been shown to have slower hippocampal growth,^21^ and a negative association between maternal stress hormone at early gestation and left hippocampal volume has been noted in children.^19^ The findings in the present study are in line with the results of these previous studies, showing that these aberrant regional brain growth disturbances likely begin in utero. This study applied noninvasive quantitative interrogation of fetal brain development in the prenatal period, which eliminated the possible influences of postpartum environmental confounders, thereby validating the association of prenatal maternal psychological distress with later brain development and neuropsychological consequences in children and adults.

The mechanisms by which maternal psychological distress affects fetal brain development remain unclear. Several mechanisms have been proposed, including increased uterine artery resistance with impaired placental perfusion and potential decreased oxygen delivery to the fetal brain^58^ as well as disrupted maternal sleep and appetite.^59^ Impaired placental function has also been implicated, including decreased placental expression of monoamine oxidase A,^60^ which may increase exposure of the fetus to 5-hydroxytryptamine, and 11β-hydroxysteroid dehydrogenase type 2,^61^ which may increase exposure to cortisol. In addition, a growing body of evidence suggests an association between prenatal maternal inflammation (interleukin 6) and altered newborn brain structure and functional connectivity.^62,63^ These data suggest a possible mediator between maternal psychosocial stress and offspring neurodevelopment, given that stress has been associated with increased inflammatory markers and altered cytokine production during pregnancy.^64,65,66,67^ Studies have demonstrated that maternal psychological distress affects DNA methylation in the glucocorticoid receptor gene (*NR3C1*) and corticotropin-releasing hormone in neonatal cord blood^68^ as well as brain-derived neurotrophic factor in infants.^69^ These data point to potential disturbances in fetal epigenetic regulation.

We showed that in vivo fetal hippocampal development was stunted by maternal psychological distress. Although previous studies showed an association between prenatal maternal psychological distress and hippocampal development in their offspring,^21,70^ to our knowledge, no studies have demonstrated these changes in the human fetus. The hippocampus plays a central role in memory and behavioral inhibition^71,72^ and contains high concentrations of corticosteroid receptors.^73^ Maternal psychological distress has been associated with epigenetic changes in neonatal cord blood, including key genes regulating the hypothalamic-pituitary-adrenal axis (ie, *NR3C1* and corticotropin-releasing hormone).^68^ Given the role of the hippocampus in stimulating and inhibiting the hypothalamic-pituitary-adrenal axis in response to stress, it is conceivable that alterations to the hypothalamic-pituitary-adrenal axis may mediate changes in the developing hippocampus. Furthermore, genetic involvement of brain-derived neurotrophic factor has been associated with variation in human hippocampal volume and function.^74,75^ These findings support the hypothesis that maternal psychological distress likely disrupts early-life hippocampal development in the human fetus.

The present study also found the presence of a prenatal hemispheric asymmetry, in which the fetal left hippocampus was substantially smaller than the right. This finding is in keeping with a previous fetal MRI study,^48^ suggesting that the asymmetric development of the hippocampi starts in utero. Our data suggested that, in human fetuses, maternal psychological distress selectively affected the left hippocampal volumetric growth more than the right. Childhood abuse–related posttraumatic stress disorder and schizophrenia have been associated with a smaller left hippocampus.^76,77^ It has been posited that the left hippocampus modulates episodic verbal memory, whereas the right hippocampus modulates spatial memory.^78^ The long-term consequences of impaired prenatal hippocampi on child outcomes remain unclear and are currently under investigation.

In addition, we report for the first time, to our knowledge, that maternal psychological distress may be associated with increased fetal local gyrification index in the frontal and temporal lobes. Studies have shown an increased gyrification index in frontal and temporal lobes in adults with schizophrenia.^79,80^ An increased gyrification index has also been reported in the frontal lobe of children with autism.^81^ These findings suggest that mental health may play a role in the complexity of brain cortical folding, and this vulnerability might increase in the frontal and temporal lobes. Conversely, studies in children have not found increased cerebral cortical gyrification after prenatal exposures to maternal mental distress.^57^ Postnatal longitudinal imaging studies are needed to confirm our initial observations.

Data from this study suggested that maternal psychological distress was associated with decreased choline, creatine, and NAA levels in the fetal brain. Fetal 1H-MRS provided a noninvasive method to study brain maturation at the biochemical level.^32^ Early metabolic alterations in the fetal brain have been shown to precede morphologic brain changes^30^ and can provide insights into the mechanisms of fetal brain insults and antecedents of injury.^32^ According to animal studies, perinatal stress-exposed rats’ offspring showed reduced NAA in the frontal cortex and hypothalamus.^26,27,28^ Decreased choline and creatine levels were noted in the left hippocampus and centrum semiovale in adults with anxiety disorder.^82,83^ Choline has a role in stem cell proliferation and apoptosis, thereby influencing brain structure and function.^84^ Both NAA, a neuronal marker, and creatine, a cellular energy currency marker, were found to increase with increasing gestational age (eTable 3 in the Supplement) and to signal brain maturation. These preliminary data suggest that altered fetal brain metabolism in the setting of maternal psychological distress may have implications for the altered fetal brain development. The long-term functional implications of these prenatal biochemical alterations are currently unknown but under investigation.

The findings of this study suggest that the prevalence of prenatal maternal psychological distress may be underestimated in healthy pregnant women of higher socioeconomic status and educational level. Maternal stress, depression, and anxiety, even if not reaching the severity of a mental disorder, were associated with altered fetal brain structure and metabolism, suggesting altered in utero programming. These findings support routine screening for prenatal psychological distress for pregnant women, even those receiving care in low-risk obstetric clinics.

### Limitations

This study has some limitations. First, questionnaires distributed earlier in gestation (ie, first trimester), and perhaps before gestation, are needed to identify the timing and onset of maternal psychological distress and its association with fetal brain development. Second, the cohort included mostly well-educated and employed women. The nature and/or severity of psychological distress in this cohort may not be representative of a broader population of pregnant women with varying sociodemographic backgrounds. In addition, the data were from a racially/ethnically diverse population, and fetal growth patterns may vary across different races/ethnicities. However, we did not find a material effect of maternal race/ethnicity on the estimates when measuring the associations between maternal psychological distress and fetal brain volumes, cortical folding, and metabolism. Moreover, the prevalence of maternal psychological distress may change with different cutoff scores. We selected cutoff scores that have been previously used for pregnant women.^37,38,40^ Because of the lack of tools for automatic segmentation of fetal brain MRI scans, we used the Draw-EM algorithm,^46^ which was originally designed for preterm brain MRI data. However, Draw-EM has been used in other studies to obtain fetal brain segmentations.^47^ After using Draw-EM, we further performed manual corrections on the initial segmentations, and the interrater reliability showed excellent agreement from the results of 2 experienced raters (ie, intraclass correlation coefficient greater than 0.95). Furthermore, because of the challenges in fetal MRI study, 8% of the MRI scans could not be used because of severe fetal motion and 19% of 1H-MRS scans were not successfully obtained; however, the percentage of lost data in this study is still similar or superior to that in other fetal MRI studies.^32,85^ Work is ongoing to develop and refine our fetal MRI techniques to increase the percentage of usable data, examine the timing of the association of maternal psychological distress with long-term cognitive and social-behavioral outcomes in children, and explore successful cognitive behavioral strategies to prevent or reduce the psychological distress in women during pregnancy and after birth.

## Conclusions

Findings of this study suggested an association between maternal psychological distress and impaired fetal hippocampal growth and brain biochemistry as well as increased fetal cortical gyrification in the frontal and temporal lobes. Postnatal imaging studies are needed to confirm the initial observations of this study.

## Notes

Supplement. **eTable 1.** Psychological Distress Scales in Pregnant Women With 2 Visits (74 Subjects) **eTable 2. **Fetal Brain Volumes, Cortical Folding Measures, and Metabolic Measures of the Overall Study Sample, and By Fetal Sex **eTable 3. **Association Between Fetal Brain Volumes/Cortical Folding/Metabolic Measures and Gestational Age **eFigure 1.** Fetal Brain Parcellation **eFigure 2. **Plots of Cortical Folding Measures on a Fetal Brain Surface (36.7 Gestational Weeks) **eFigure 3. **Fetal Brain MRS Measures **eFigure 4. **Flow Diagram Summarizing Our Subject Recruitment in This Study
