# Supplementary material for: Association of Prenatal Maternal Psychological Distress With Fetal Brain Growth, Metabolism, and Cortical Maturation
Source: JAMA Netw Open. 2020 Jan 29;3(1):e1919940. doi: 10.1001/jamanetworkopen.2019.19940 (PMC6991285; doi:10.1001/jamanetworkopen.2019.19940)
Supplement: Supplement. — eTable 1. Psychological Distress Scales in Pregnant Women With 2 Visits (74 Subjects) eTable 2. Fetal Brain Volumes, Cortical Folding Measures, and Metabolic Measures of the Overall Study Sample, and By Fetal Sex eTable 3. Association Between Fetal Brain Volumes/Cortical Folding/Metabolic Measures and Gestational Age eFigure 1. Fetal Brain Parcellation eFigure 2. Plots of Cortical Folding Measures on a Fetal Brain Surface (36.7 Gestational Weeks) eFigure 3. Fetal Brain MRS Measures eFigure 4. Flow Diagram Summarizing Our Subject Recruitment in This Study [file jamanetwopen-3-e1919940-s001.pdf]

Supplementary Online Content

Wu Y, Lu Y-C, Jacobs M, et al. Association of prenatal maternal psychological distress with fetal brain growth, metabolism, and cortical maturation. *JAMA Netw Open.* 2020;3(1): e1919940. doi:10.1001/jamanetworkopen.2019.19940

eTable 1. Psychological Distress Scales in Pregnant Women With 2 Visits (74 Subjects)

eTable 2. Fetal Brain Volumes, Cortical Folding Measures, and Metabolic Measures of the Overall Study Sample, and By Fetal Sex

eTable 3. Association Between Fetal Brain Volumes/Cortical Folding/Metabolic Measures and Gestational Age

eFigure 1. Fetal Brain Parcellation

eFigure 2. Plots of Cortical Folding Measures on a Fetal Brain Surface (36.7 Gestational Weeks)

eFigure 3. Fetal Brain MRS Measures

eFigure 4. Flow Diagram Summarizing Our Subject Recruitment in This Study

This supplementary material has been provided by the authors to give readers additional information about their work.

eTable 1. Psychological Distress Scales in Pregnant Women With 2 Visits (74 Subjects)

Mean (SD)	1st visit	2nd visit	P value
SSAI	29.68 (8.59)	28.51 (7.54)	.19
STAI	31 (8.49)	29.71 (7.43)	.01*
PSS	10.99 (5.28)	9.51 (5.55)	<.001*
EPDS	4.28 (3.26)	4.04 (3.52)	.39

Abbreviations: SSAI, Spielberger State Anxiety Inventory; STAI, Spielberger Trait Anxiety Inventory; PSS, Perceived Stress Scale; EPDS, Edinburgh Postnatal Depression Scale.
P value based on paired t-test
* Significant after adjusting for multiple testing

©2020 Wu Y et al. *JAMA Network Open*

eTable 2. Fetal Brain Volumes, Cortical Folding Measures, and Metabolic Measures of the Overall Study Sample, and By Fetal Sex

Mean	Overall	Female	Male	P Value
**Brain Volumes (cm^3)** **(119 subjects: 52 females, 67 males)**				
Total brain	203.70	197.88	208.16	<.001*
Cortical grey matter	66.66	64.44	68.41	.007*
White matter	108.77	105.68	111.20	.008*
Deep grey matter	18.36	17.93	18.70	.002*
Cerebellum	10.22	10.15	10.28	.54
Brainstem	4.38	4.30	4.45	.01*
Left hippocampus	0.53	0.52	0.54	.12
Right hippocampus	0.56	0.55	0.57	.10
**Cortical Folding Measures (whole brain)** **(99 subjects: 43 females, 56 males)**				
Local gyrification index	1.43	1.44	1.43	.38
Sulcal depth (mm)	1.97	1.97	1.97	.99
Curvedness (mm^{-1})	0.22	0.22	0.22	.50
**Metabolic Measures** **(100 subjects: 48 females, 52 males)**				
N-acetylaspartate	3.61	3.50	3.71	.14
Creatine	3.01	2.95	3.06	.18
Choline	2.46	2.49	2.45	.61
Results of least squares means estimates from generalized estimating equations, controlling for gestational age at MRI<br>* Significant after adjusting for multiple testing				

eTable 3. Association Between Fetal Brain Volumes/Cortical Folding/Metabolic Measures and Gestational Age

Measures	Overall		Female		Male		Sex/GA effect
	β	P Value	β	P Value	β	P Value	P Value
**Brain Volumes (cm^3) (119 subjects: 52 females, 67 males)**							
Total brain	17.80	<.001	16.93	<.001	18.50	<.001	<.001*
Cortical grey matter	5.86	<.001	5.50	<.001	6.20	<.001	.008*
White matter	9.14	<.001	8.73	<.001	9.50	<.001	.04
Deep grey matter	1.33	<.001	1.28	<.001	1.37	<.001	.02
Cerebellum	1.20	<.001	1.18	<.001	1.21	<.001	.50
Brainstem	0.29	<.001	0.28	<.001	0.29	<.001	.09
Left hippocampus	0.039	<.001	0.038	<.001	0.040	<.001	.28
Right hippocampus	0.040	<.001	0.039	<.001	0.041	<.001	.23
**Cortical Folding Measures (whole brain) (99 subjects: 43 females, 56 males)**							
Local gyrification index	0.02	<.001	0.02	<.001	0.03	<.001	.57
Sulcal depth (mm)	0.16	<.001	0.17	<.001	0.16	<.001	.87
Curvedness (mm^{-1})	0.01	<.001	0.009	<.001	0.01	<.001	.36
**Metabolic Measures (100 subjects: 48 females, 52 males)**							
N-acetylaspartate	0.20	<.001	0.20	<.001	0.19	<.001	.49
Creatine	0.10	<.001	0.11	<.001	0.09	<.001	.56
Choline	0.01	.10	0.02	.05	0.004	.69	.20

Abbreviations: GA, gestational age.
Results based on generalized estimating equations
* Significant after adjusting for multiple testing

©2020 Wu Y et al. *JAMA Network Open*

eFigure 1. Fetal Brain Parcellation

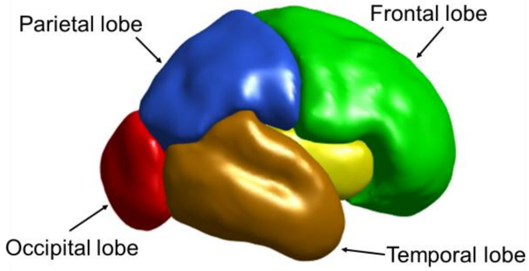

Parcellated frontal (green), parietal (blue), temporal (brown), and occipital (red) lobes of a fetal brain at 28.3 gestational weeks.

©2020 Wu Y et al. *JAMA Network Open*

eFigure 2. Plots of Cortical Folding Measures on a Fetal Brain Surface (36.7 Gestational Weeks)

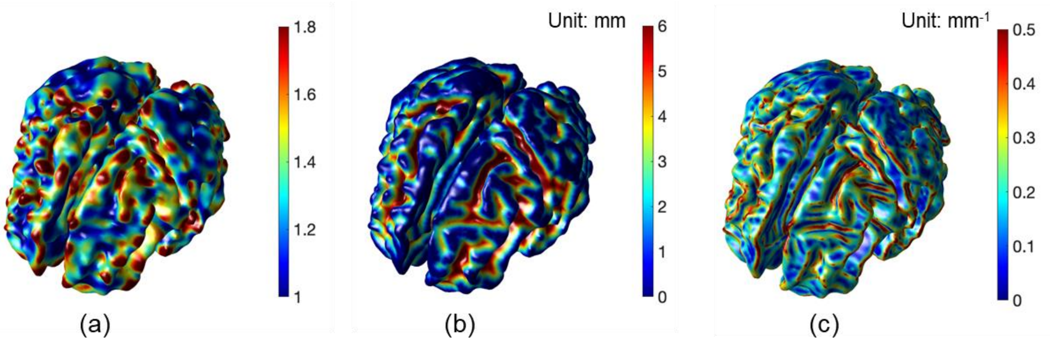

(a) Local gyrification index; (b) Sulcal depth; (c) Curvedness.

©2020 Wu Y et al. *JAMA Network Open*

eFigure 3. Fetal Brain MRS Measures

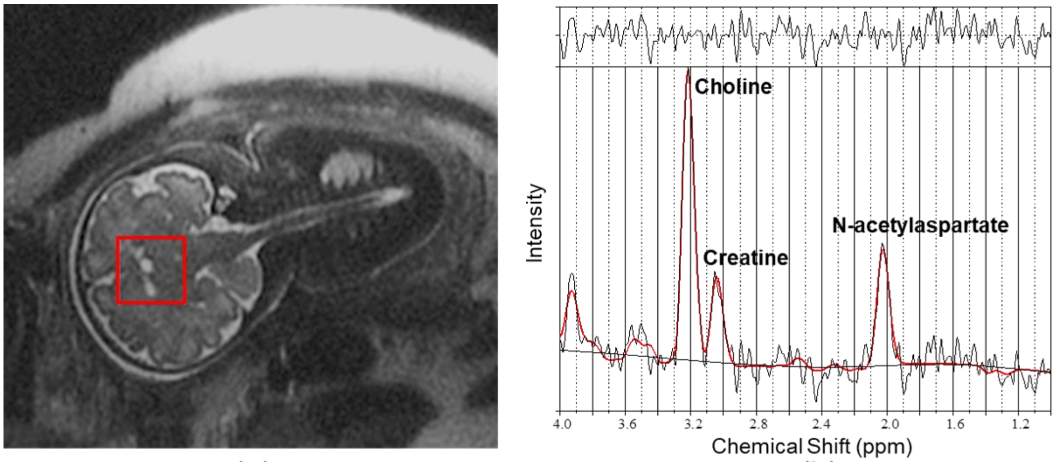

(a) (b)

(a) the spectral voxel was placed in the center of fetal brain using the anatomical image as a guidance; (b) representative choline, creatine, and N-acetylaspartate metabolites at 35 gestational weeks.

©2020 Wu Y et al. *JAMA Network Open*

eFigure 4. Flow Diagram Summarizing Our Subject Recruitment in This Study

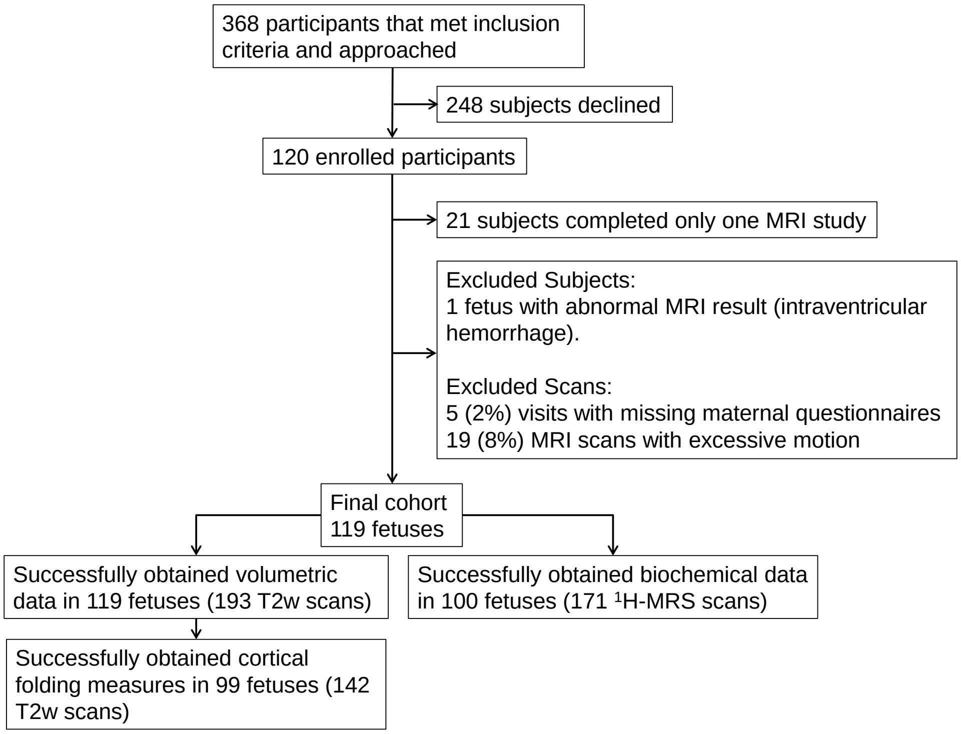

©2020 Wu Y et al. *JAMA Network Open*
